# Predictive Performance of Simplified First-Trimester Placental Volume Estimation Combined with Uterine Artery Doppler and Maternal Serum Biomarkers for Preeclampsia and Fetal Growth Restriction: A Retrospective Cohort Study

**DOI:** 10.3390/metabo16080571

**Published:** 2026-08-12

**Authors:** Serem Kel Ilgın, Mehmet Nuri Duran, Süreyya Sarıdaş Demir, Bülent Demir

**Affiliations:** 1 Biga State Hospital, Çanakkale 17000, Turkey; 2 Ezine State Hospital, Çanakkale 17000, Turkey; 3 Department of Perinatology, Private Clinic, Çanakkale 17000, Turkey; 4 Department of Obstetrics and Gynecology, Çanakkale Onsekiz Mart University, Çanakkale 17000, Turkey

**Keywords:** placental volume, preeclampsia, fetal growth restriction, early prediction, placental dysfunction, uterine artery Doppler, PAPP-A, free β-hCG, first trimester

## Abstract

**Highlights:**

**What are the main findings?**

- Placental volume alone poorly predicted preeclampsia and fetal growth restriction.
- Lower free β-hCG remained independently associated with fetal growth restriction.

**What are the implications of the main findings?**

- Internal validation markedly reduced the apparent performance of the combined model.
- Currently available first-trimester markers are unlikely to suffice as stand-alone screening tests.

**Abstract:**

**Background**: Preeclampsia and fetal growth restriction (FGR) are major obstetric complications originating from abnormal placental development during early pregnancy. Early identification of pregnancies at increased risk remains challenging despite the availability of several first-trimester biochemical and biophysical markers. **Objective**: This study aimed to evaluate the predictive value of first-trimester placental volume, bilateral uterine artery Doppler pulsatility indices (UtA-PI), pregnancy-associated plasma protein-A (PAPP-A), and free β-human chorionic gonadotropin (free β-hCG) for the subsequent development of preeclampsia and FGR. **Methods**: This retrospective cohort study included 251 singleton pregnancies that underwent routine first-trimester aneuploidy screening. Placental volume was estimated using a standardized two-dimensional ultrasonographic method; bilateral uterine artery Doppler examinations were performed according to Fetal Medicine Foundation recommendations, and maternal serum PAPP-A and free β-hCG values were recorded as multiples of the median. Receiver operating characteristic (ROC) analysis, multivariable logistic regression, and leave-one-out cross-validation were performed to evaluate predictive performance. **Results**: Preeclampsia and FGR developed in 8 (3.2%) and 29 (11.6%) pregnancies, respectively. None of the investigated biochemical or biophysical markers differed significantly between affected and unaffected pregnancies. Individual biomarkers demonstrated poor discriminatory performance, with AUC values ranging from 0.50 to 0.64. Following adjustment for maternal characteristics, only lower maternal free β-hCG remained independently associated with FGR (adjusted OR 0.49, 95% CI 0.25–0.96; *p* = 0.038). The apparent performance of the combined prediction model was not maintained following internal validation, indicating substantial model overfitting. **Conclusions:** First-trimester placental volume, uterine artery Doppler indices, PAPP-A, and free β-hCG demonstrated limited predictive performance for preeclampsia and FGR in this cohort. These findings suggest that currently available first-trimester biomarkers are insufficient as stand-alone screening tools and support the development of multimodal prediction strategies integrating biochemical, biophysical, angiogenic, and molecular biomarkers rather than relying on isolated first-trimester markers.

## 1. Introduction

Hypertensive disorders of pregnancy and fetal growth restriction (FGR) remain among the leading causes of maternal and perinatal morbidity and mortality worldwide [1]. Preeclampsia complicates approximately 2–5% of pregnancies, whereas FGR affects nearly 5–10% of singleton pregnancies and accounts for a substantial proportion of stillbirths, neonatal intensive care admissions, and long-term neurodevelopmental impairment [2,3,4,5]. Although their clinical manifestations differ, both conditions originate primarily from abnormal placentation during early pregnancy [6,7].

Normal placental development begins shortly after implantation and is characterized by trophoblastic invasion of the maternal spiral arteries and progressive remodeling of the uteroplacental circulation into a low-resistance, high-capacitance vascular system. Failure of this physiological transformation results in placental hypoperfusion, oxidative stress, endothelial dysfunction, and impaired fetoplacental development. Consequently, abnormal placental development has been recognized as the central pathological mechanism underlying both preeclampsia and fetal growth restriction [4,8,9].

Because these pathological processes originate during the first trimester, considerable attention has focused on identifying early biomarkers capable of predicting subsequent placental dysfunction. Current first-trimester screening strategies incorporate maternal characteristics together with biochemical markers, including pregnancy-associated plasma protein-A (PAPP-A) and free β-human chorionic gonadotropin (free β-hCG), as well as biophysical parameters such as uterine artery Doppler velocimetry [10,11,12,13]. Although these markers improve prediction when combined in sophisticated screening algorithms, their performance remains insufficient for universal clinical implementation, particularly in low-risk populations.

Placental volume has emerged as another promising indicator of early placental development. Several studies have demonstrated that pregnancies subsequently complicated by preeclampsia or fetal growth restriction tend to exhibit smaller placental volumes during the first trimester [14,15,16,17,18]. Since placental growth directly reflects trophoblastic proliferation and villous branching, quantitative assessment of placental volume may provide a direct measure of placental development before clinical disease becomes apparent.

Most previous investigations have measured placental volume using three-dimensional (3D) ultrasonography combined with Virtual Organ Computer-aided Analysis (VOCAL). Although this technique provides accurate volumetric assessment, its routine clinical application is limited by the need for dedicated software, prolonged acquisition time, specialized operator training, and reduced availability in many obstetric centers.

To overcome these limitations, simplified two-dimensional (2D) placental volume estimation methods have been developed. This approach calculates placental volume using three easily obtainable sonographic measurements—maximum placental width (W), maximum placental height (H), and maximum placental thickness (T)—which can be acquired rapidly during routine first-trimester ultrasound examinations without additional software or specialized equipment. Previous studies have suggested acceptable agreement between this simplified method and 3D placental volumetry while considerably improving feasibility in daily clinical practice.

However, evidence regarding the predictive performance of 2D placental volume estimation remains limited, and studies simultaneously evaluating placental volume together with established biochemical and Doppler markers are scarce. Furthermore, most published studies have involved relatively small populations and have produced inconsistent results regarding the prediction of preeclampsia and fetal growth restriction [19,20,21].

Despite considerable progress in first-trimester screening, there remains no universally accepted model combining simple ultrasonographic and routinely available biochemical markers for the accurate prediction of placental dysfunction in low-risk singleton pregnancies. Therefore, the primary aim of the present study was to investigate the predictive value of first-trimester placental volume measured using a simplified two-dimensional ultrasonographic technique, together with maternal serum free β-hCG, PAPP-A, and bilateral uterine artery Doppler pulsatility indices, for the subsequent development of preeclampsia and fetal growth restriction. In addition, we evaluated the diagnostic performance of these markers individually and in combination using multivariable prediction models and internal validation techniques.

## 2. Materials and Methods

### 2.1. Study Design and Population

This retrospective cohort study was conducted at the Obstetrics and Gynecology Outpatient Clinic of Çanakkale Onsekiz Mart University Health Practice and Research Hospital. Pregnant women who underwent routine first-trimester aneuploidy screening were screened for eligibility.

Initially, 255 singleton pregnancies were identified. Four women were excluded because complete pregnancy outcome data were unavailable, leaving 251 pregnancies for the final analysis. Women aged ≥18 years with viable singleton pregnancies between 11 + 0 and 13 + 6 weeks of gestation were eligible for inclusion. Multiple pregnancies, major fetal structural abnormalities diagnosed during pregnancy, chromosomal abnormalities, pregnancies ending in miscarriage before fetal viability, and cases with incomplete clinical records were excluded.

The study was conducted in accordance with the Declaration of Helsinki and was approved by the Clinical Research Ethics Committee of Çanakkale Onsekiz Mart University (Approval No. 2022-03; 2 February 2022).

### 2.2. Clinical Data Collection

Maternal demographic and clinical characteristics were retrieved retrospectively from the hospital electronic medical records. The following variables were recorded: maternal age, maternal weight, gravidity, parity, smoking status, pre-existing maternal disease, mode of delivery, placental volume, right and left uterine artery pulsatility indices, free β-hCG (multiples of the median, MoM), and PAPP-A (MoM). Pregnancy outcomes were obtained after delivery using the institutional electronic database.

### 2.3. Ultrasound Examination

All first-trimester ultrasound examinations were performed transabdominally using a GE Voluson S6 ultrasound system (GE Healthcare, Zipf, Austria) during routine aneuploidy screening. Gestational age was determined according to crown–rump length measurements following current international recommendations. Placental location, fetal viability, and routine first-trimester anatomical assessment were completed before placental measurements.

### 2.4. Placental Volume Measurement

**Figure 1:**
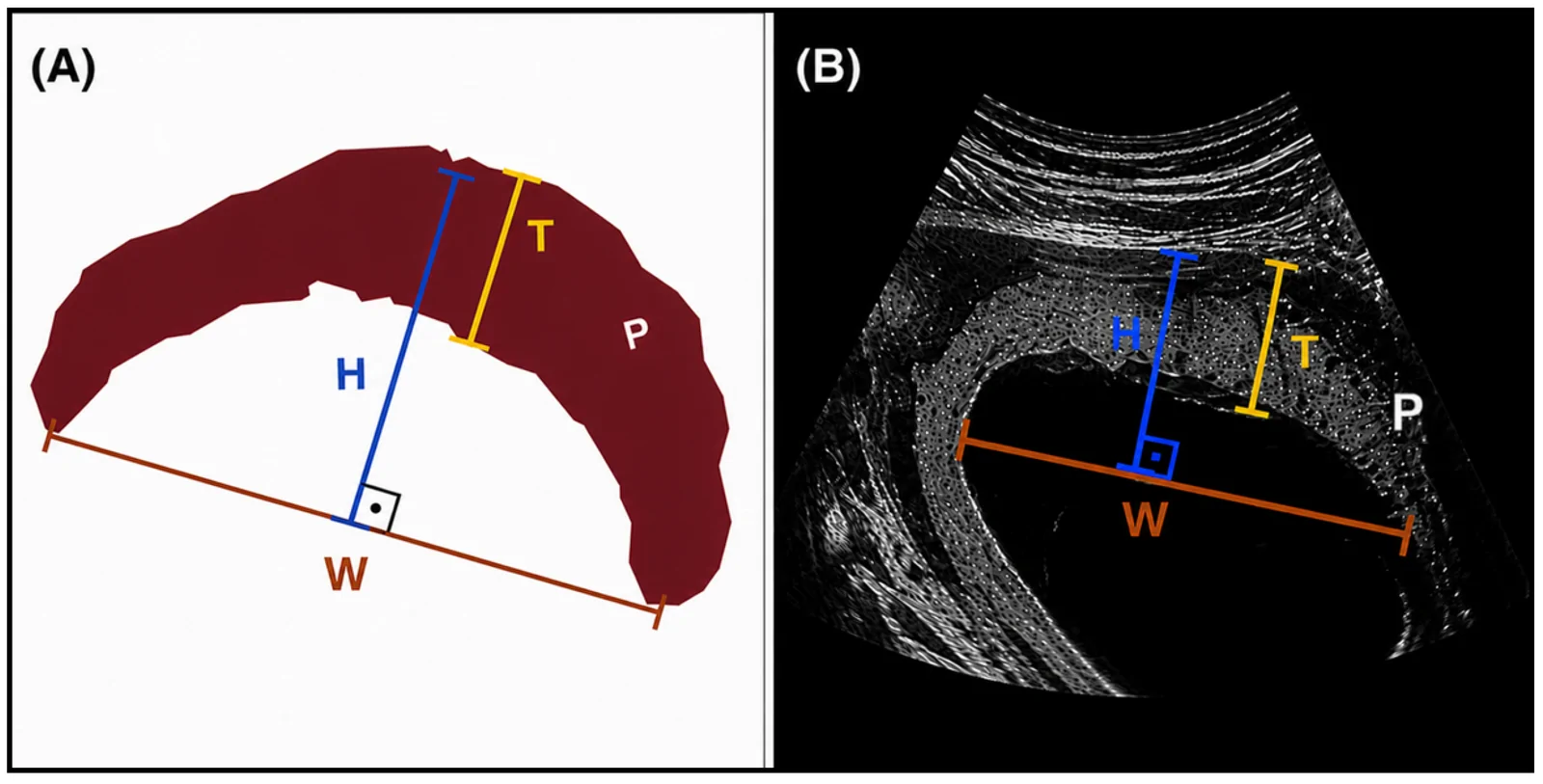
Two-dimensional placental volume measurement: (**A**) Schematic illustration and (**B**) representative first-trimester ultrasound image showing the placenta (P), with placental width (W), height (H), and thickness (T) measured using a standardized two-dimensional ultrasonographic technique; placental volume was subsequently calculated using the published mathematical formula.

Placental volume was estimated using a standardized two-dimensional ultrasonographic technique previously described by Sonek et al. [22]. After obtaining the midsagittal view of the placenta, three placental dimensions were measured (Figure 1): maximum placental width (W), maximum placental height (H), and maximum placental thickness (T). Maximum placental width was defined as the longest distance between both placental edges along the chorionic plate. Placental height was measured perpendicular (90°) to the width, from the chorionic plate to the uteroplacental interface. Placental thickness was measured at the point of maximum placental thickness along the same perpendicular axis. Placental volume was calculated using the following equation:

V = (π × T/6) × [4H(W − T) + W(W − 4T) + 4T^2^] (1)

where W represents placental width, H placental height, and T placental thickness. All measurements were obtained during the same examination before Doppler assessment.

### 2.5. Uterine Artery Doppler Examination

**Figure 2:**
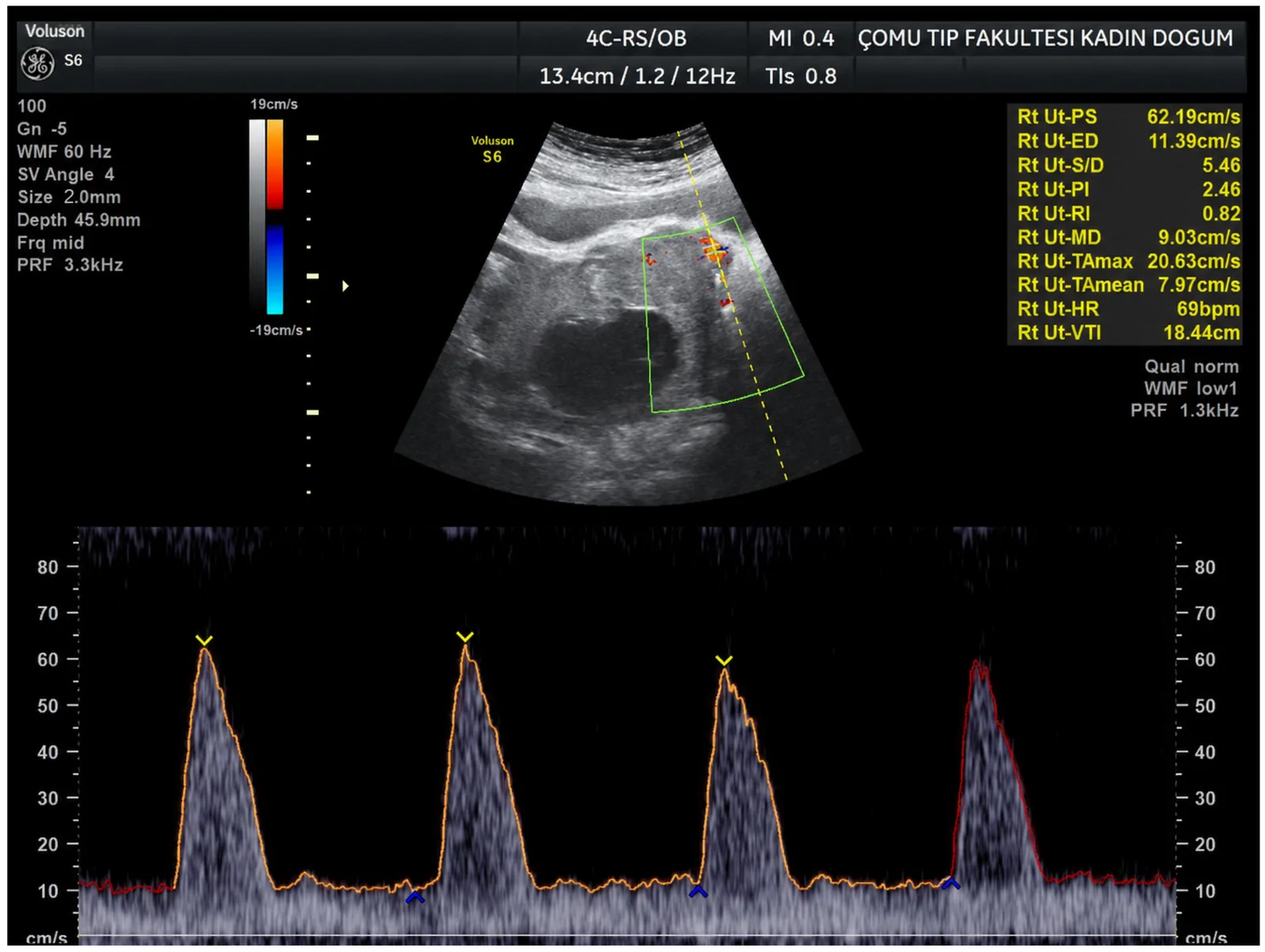
Representative bilateral uterine artery Doppler examination performed according to the Fetal Medicine Foundation protocol. The three orange-traced waveforms represent the measured cycles used for pulsatility index calculation; the additional (red-traced) waveform is an adjacent, unmeasured cardiac cycle automatically displayed by the ultrasound system. The green box denotes the Doppler sample gate positioned over the uterine artery, the yellow dashed line indicates the angle-correction line used to align the insonation angle with the vessel, and the yellow and blue chevrons mark the automatically identified systolic peak and end-diastolic points, respectively, used for pulsatility index calculation.

Bilateral uterine artery Doppler velocimetry was performed according to the recommendations of the Fetal Medicine Foundation [23]. Color Doppler imaging was used to identify the uterine arteries at the level of the internal cervical os. Pulsed-wave Doppler recordings were obtained with an insonation angle < 30°, a sample volume of 2 mm, and a peak systolic velocity > 60 cm/s. Three consecutive similar waveforms were obtained before pulsatility index (PI) measurements were recorded (Figure 2). For each uterine artery, the pulsatility index was calculated as the average of three technically satisfactory, consecutive similar waveforms, and the mean of the right and left uterine artery PI values was used in the subsequent statistical analyses.

### 2.6. Outcome Definitions

Preeclampsia was defined according to the American College of Obstetricians and Gynecologists (ACOG) criteria [24] as new-onset hypertension after 20 weeks’ gestation associated with proteinuria or maternal end-organ dysfunction. Fetal growth restriction was defined as an estimated fetal weight below the 10th percentile for gestational age using contemporary fetal growth charts [25], consistent with the Delphi consensus definition of FGR [26].

### 2.7. Statistical Analysis

Statistical analyses were performed using IBM SPSS Statistics version 26.0 (IBM Corp., Armonk, NY, USA) together with Python 3.12 (Python Software Foundation, Wilmington, DE, USA). Continuous variables were assessed for normality using the Shapiro–Wilk test. Normally distributed variables were compared using the independent-sample *t*-test, whereas non-normally distributed variables were analyzed using the Mann–Whitney U test. Categorical variables were compared using Pearson’s χ^2^ test or Fisher’s exact test where appropriate. Fewer than 2% of eligible records had missing data; a complete-case analysis was therefore performed. Multicollinearity among predictors in the multivariable models was assessed using variance inflation factors (VIFs), all of which were below 1.2, indicating no significant collinearity.

Receiver operating characteristic (ROC) curve analysis was performed to evaluate the predictive performance of each biomarker. Areas under the ROC curve (AUCs) with 95% confidence intervals were calculated, and optimal cut-off values were determined using the Youden index. Variables associated with pregnancy outcomes were further evaluated using univariable and multivariable logistic regression analyses after adjustment for maternal age, maternal weight, parity, and smoking status. To assess the robustness of multiple comparisons, *p*-values were additionally corrected using the Benjamini–Hochberg false-discovery-rate procedure. Internal validation of the multivariable prediction models was performed using leave-one-out cross-validation (LOOCV) together with bootstrap optimism correction. Two-sided *p*-values < 0.05 were considered statistically significant.

## 3. Results

### 3.1. Study Population

**Figure 3:**
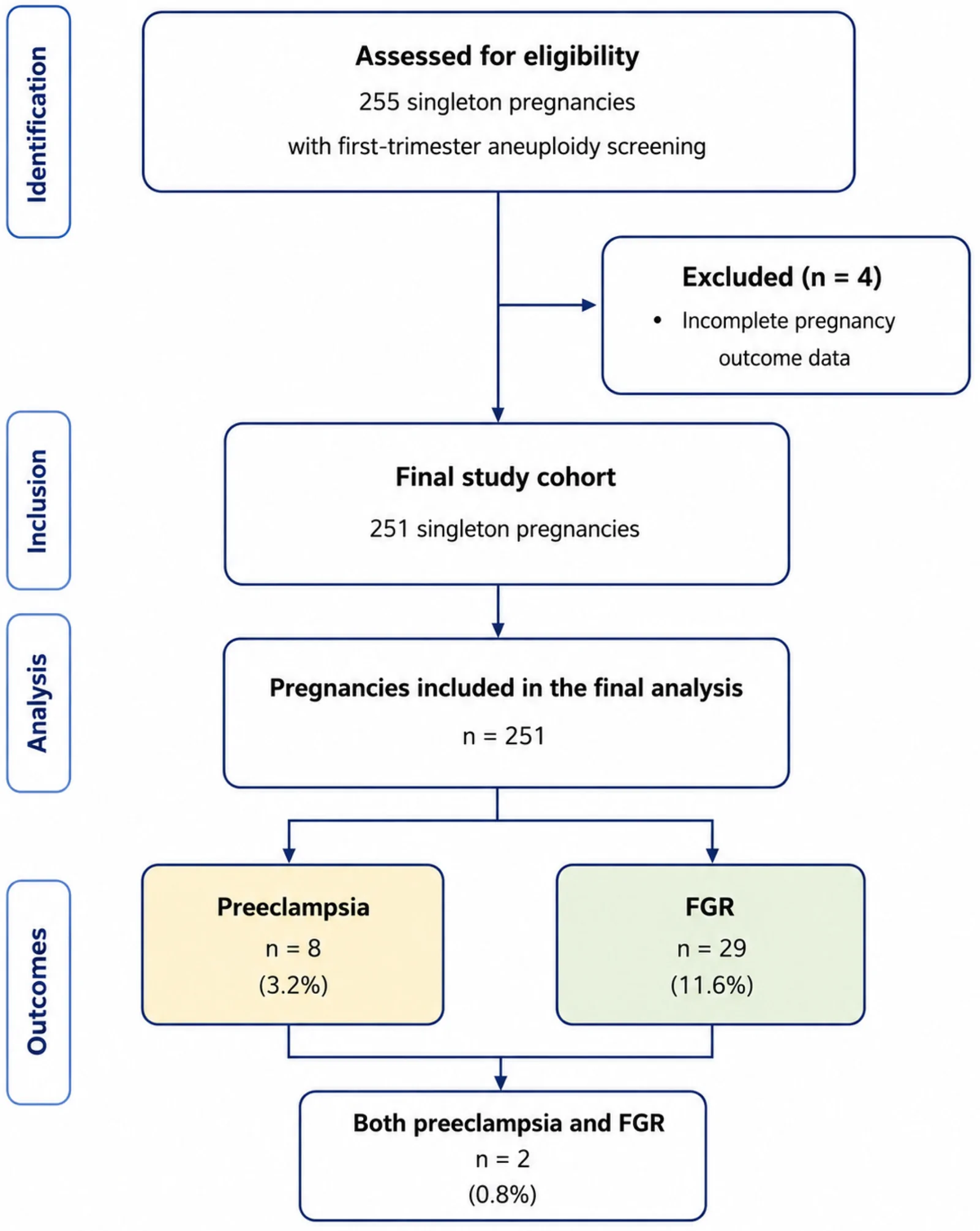
Study flow diagram of the included pregnancies.

A total of 255 singleton pregnancies were initially screened for eligibility. Four women were excluded because complete pregnancy outcome data were unavailable, leaving 251 pregnancies for the final analysis (Figure 3). Among the included pregnancies, preeclampsia developed in 8 women (3.2%), whereas fetal growth restriction (FGR) occurred in 29 pregnancies (11.6%); two pregnancies were complicated by both conditions. Baseline maternal and pregnancy characteristics are summarized in Table 1. The mean maternal age was 27.2 ± 5.3 years, and the mean maternal weight was 65.6 ± 12.5 kg. Approximately half of the study population was nulliparous (50.6%), 9.2% of women reported smoking during pregnancy, and 7.6% had a pre-existing maternal medical disorder. The overall cesarean delivery rate was 52.2%.

### 3.2. Comparison of First-Trimester Markers According to Pregnancy Outcome

First-trimester biochemical and biophysical markers according to pregnancy outcome are presented in Table 2. Women who subsequently developed preeclampsia demonstrated numerically lower placental volume and PAPP-A values together with slightly higher uterine artery pulsatility indices compared with unaffected pregnancies; however, none of these differences reached statistical significance.

Similarly, pregnancies complicated by fetal growth restriction exhibited lower median placental volume and lower free β-hCG concentrations than uncomplicated pregnancies. Although free β-hCG approached statistical significance (*p* = 0.052), no statistically significant differences were identified for placental volume, uterine artery Doppler indices, or PAPP-A. Following adjustment for multiple comparisons using the Benjamini–Hochberg procedure, none of the investigated biomarkers remained statistically significant. Overall, these findings indicate that individual first-trimester biochemical and biophysical markers showed limited ability to distinguish pregnancies that subsequently developed placental dysfunction.

### 3.3. Diagnostic Performance of Individual Biomarkers

**Figure 4:**
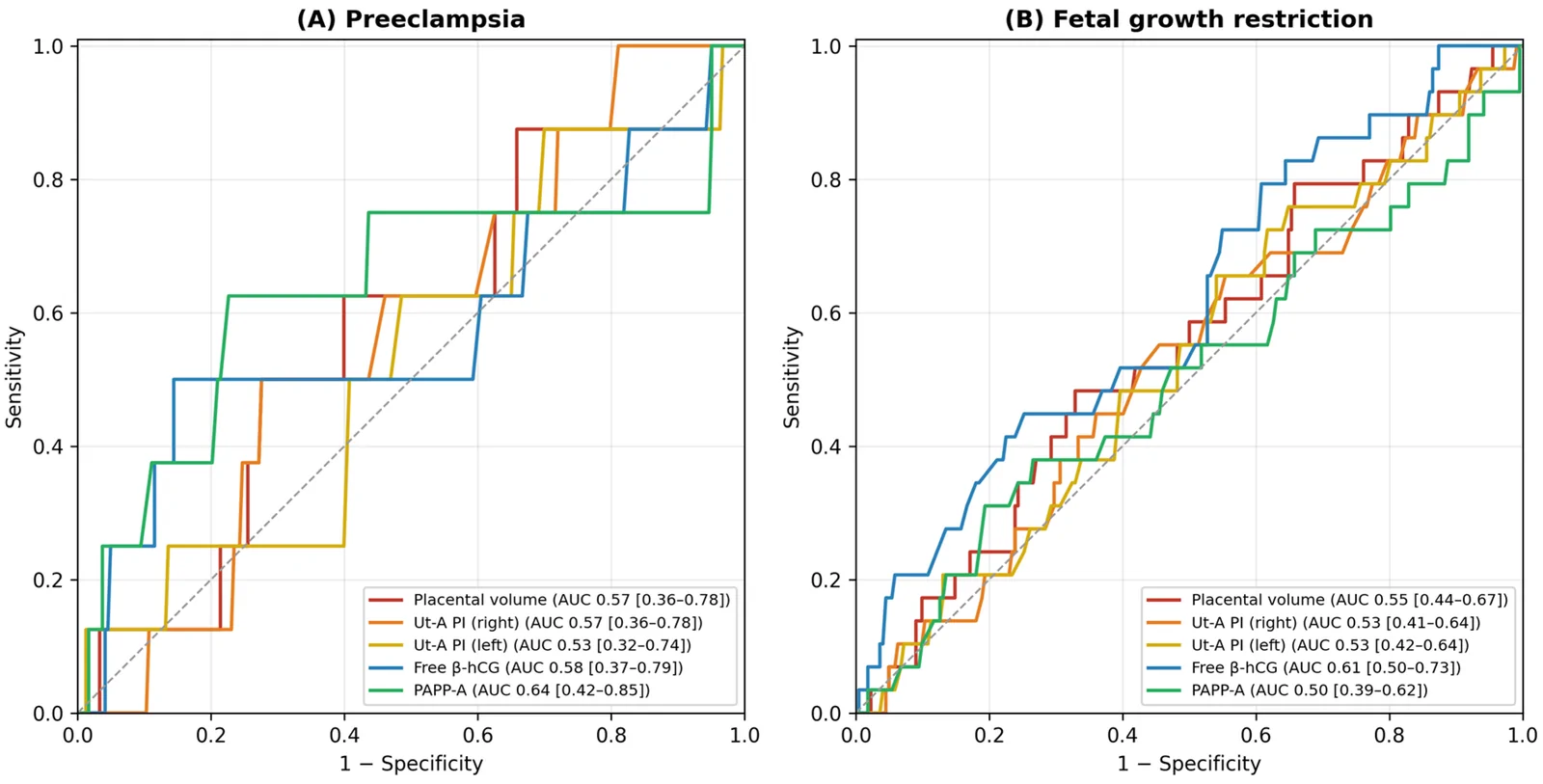
Receiver operating characteristic (ROC) curves of first-trimester biochemical and biophysical markers for predicting (**A**) preeclampsia and (**B**) fetal growth restriction. Areas under the curve (AUCs) with 95% confidence intervals are shown in the legend; the dashed diagonal line denotes no discrimination (AUC 0.50).

Receiver operating characteristic (ROC) curve analysis was performed to evaluate the discriminatory performance of each individual biomarker for predicting preeclampsia and fetal growth restriction (Figure 4). For preeclampsia, the area under the ROC curve ranged from 0.53 to 0.64, indicating poor discriminatory performance. Similarly, ROC analysis for fetal growth restriction demonstrated AUC values ranging between 0.50 and 0.61, with confidence intervals overlapping the reference value of 0.50 for all investigated markers. Placental volume demonstrated only modest discrimination for both outcomes, while bilateral uterine artery Doppler indices showed similarly limited predictive performance. Among the biochemical markers, free β-hCG produced the highest AUC for fetal growth restriction; however, its discriminatory ability remained insufficient for clinical application. Overall, none of the investigated biomarkers demonstrated acceptable diagnostic performance when evaluated individually.

### 3.4. Logistic Regression Analysis

To determine whether first-trimester markers were independently associated with adverse pregnancy outcomes, logistic regression analyses were performed after adjustment for maternal age, maternal weight, parity, and smoking status (Table 3). For preeclampsia, none of the investigated biochemical or biophysical markers remained independently associated with disease development. For fetal growth restriction, lower maternal free β-hCG remained independently associated with the outcome after adjustment for maternal characteristics (adjusted OR 0.49, 95% CI 0.25–0.96, *p* = 0.038). Placental volume, bilateral uterine artery Doppler pulsatility indices, and PAPP-A did not demonstrate independent predictive value for either outcome. These findings suggest that the predictive contribution of individual first-trimester biomarkers is limited after accounting for established maternal risk factors.

### 3.5. Performance of the Combined Prediction Model

To investigate whether combining multiple first-trimester markers improved predictive performance, a multivariable logistic regression model incorporating placental volume, bilateral uterine artery Doppler indices, free β-hCG, and PAPP-A was developed. The apparent discriminatory performance of the combined model yielded an AUC of 0.72 for preeclampsia and 0.65 for fetal growth restriction. However, internal validation using leave-one-out cross-validation demonstrated substantial model optimism, with validated AUC values decreasing to 0.20 for preeclampsia and 0.54 for fetal growth restriction. Similarly, bootstrap optimism correction reduced the predictive performance of the combined model to values close to random classification. Sensitivity analyses excluding women with additional maternal disease or an implausible Doppler measurement produced comparable results. Overall, these findings indicate that combining currently available first-trimester biochemical and biophysical markers does not provide clinically meaningful prediction of placental dysfunction within this cohort.

## 4. Discussion

The present study evaluated the predictive performance of first-trimester placental volume, bilateral uterine artery Doppler pulsatility indices, maternal serum PAPP-A, and free β-hCG for the subsequent development of preeclampsia and fetal growth restriction. Overall, none of the investigated biomarkers showed clinically meaningful predictive ability when evaluated individually or as part of a combined prediction model.

Although pregnancies complicated by placental dysfunction generally exhibited lower placental volume and lower biochemical marker levels together with slightly higher uterine artery Doppler indices, these differences were insufficient to provide clinically useful discrimination, and all areas under the ROC curve were close to 0.50.

Placental dysfunction represents the fundamental pathological mechanism underlying both preeclampsia and fetal growth restriction. Abnormal trophoblastic invasion during the first trimester results in incomplete remodeling of the maternal spiral arteries, persistent high-resistance uteroplacental circulation, placental hypoperfusion, oxidative stress, and endothelial dysfunction. These early pathological changes precede the clinical manifestations of both disorders by several months and therefore constitute the rationale for first-trimester screening strategies.

Placental volume has attracted increasing attention as a direct sonographic marker of placental development. Since placental growth reflects trophoblastic proliferation and villous branching, reduced placental volume has been proposed as an indirect indicator of impaired placentation. Several investigators have reported significantly smaller first-trimester placental volumes in pregnancies that subsequently develop preeclampsia or fetal growth restriction; however, other studies have demonstrated only modest predictive performance, particularly when placental volume is used as an isolated screening parameter [14,15,21,27].

Our findings are consistent with the latter studies. Although placental volume tended to be lower in pregnancies complicated by preeclampsia and fetal growth restriction, the observed differences were not statistically significant, and the discriminatory performance remained poor. These findings suggest that placental size alone does not adequately reflect the complex biological mechanisms responsible for placental dysfunction. Functional abnormalities involving placental perfusion, angiogenesis, oxidative stress, and endothelial activation may precede measurable structural alterations during early gestation.

Similarly, uterine artery Doppler assessment has been extensively investigated as an early marker of impaired placentation. Increased uterine artery pulsatility indices reflect increased vascular resistance resulting from incomplete spiral artery remodeling. Nevertheless, numerous studies have shown that first-trimester uterine artery Doppler has only moderate predictive performance when used alone. Current international screening algorithms therefore recommend combining uterine artery Doppler with maternal characteristics and biochemical markers rather than using Doppler measurements in isolation [28]. The present findings support this concept, as bilateral uterine artery pulsatility indices showed limited discriminatory ability for both outcomes.

Among the biochemical markers, PAPP-A and free β-hCG are routinely measured during first-trimester aneuploidy screening and have been evaluated extensively as surrogate markers of placental function. Low maternal serum PAPP-A has consistently been associated with placental insufficiency and adverse pregnancy outcomes, whereas evidence regarding free β-hCG has been less consistent; in a recent first-trimester combined-marker study, low PAPP-A was the strongest early predictor of FGR, while placental volume again showed only limited discrimination (AUC 0.57), closely mirroring the present findings [21]. In the present study, neither biochemical marker demonstrated clinically meaningful predictive performance. However, lower maternal free β-hCG remained independently associated with fetal growth restriction after adjustment for maternal characteristics. Although biologically plausible, this isolated association should be interpreted cautiously because the effect size was modest and did not translate into clinically useful discrimination.

One of the most important findings of the present study is that combining multiple first-trimester biomarkers did not substantially improve predictive performance after internal validation. The apparent improvement observed in the initial multivariable model disappeared following leave-one-out cross-validation, indicating considerable model optimism and overfitting. This finding emphasizes an important methodological issue frequently overlooked in prediction studies: apparent model performance may substantially overestimate clinical usefulness when the number of outcome events is limited. Internal validation therefore represents an essential component of prediction model development and should be routinely incorporated into future obstetric biomarker studies.

Several factors may explain the limited predictive performance observed in the present study. First, placental dysfunction is a multifactorial biological process involving complex interactions among maternal cardiovascular adaptation, placental angiogenesis, immune regulation, oxidative stress, and genetic susceptibility; it is therefore unlikely that any single biochemical or ultrasonographic marker can adequately capture this complexity. Second, placental morphological changes measurable by ultrasonography may occur relatively late compared with the underlying molecular abnormalities responsible for disease development. Finally, substantial biological heterogeneity exists within both preeclampsia and fetal growth restriction, suggesting that different pathogenic pathways may predominate among individual pregnancies.

### 4.1. Strengths

The present study has several important strengths. To our knowledge, this is among the few studies simultaneously evaluating simplified two-dimensional placental volume estimation together with routine biochemical markers and uterine artery Doppler parameters within the same prediction model. First, it simultaneously evaluated biochemical and biophysical first-trimester markers within the same patient cohort, allowing direct comparison of their relative predictive contributions. Second, placental volume was obtained using a standardized, reproducible two-dimensional technique that is feasible in routine practice and does not require dedicated three-dimensional software. Third, in addition to conventional ROC analysis and multivariable logistic regression, contemporary statistical safeguards—correction for multiple comparisons, confounder adjustment, and internal validation using leave-one-out cross-validation and bootstrap optimism correction—were applied, providing a more realistic estimate of clinical performance than apparent accuracy alone. Fourth, the transparent reporting of negative and internally validated findings adds to the limited literature that critically appraises the true predictive value of these widely used markers.

### 4.2. Limitations

Several limitations should also be acknowledged. First, the retrospective design may have introduced selection and information bias. Second, the single-center setting may limit the generalizability of the findings to other populations and healthcare settings. Third, the relatively small number of preeclampsia cases reduced statistical power and increased the risk of model overfitting. Fourth, no external validation cohort was available to confirm the internal validation results. Fifth, placental volume was estimated using a two-dimensional technique rather than three-dimensional volumetric analysis, which may introduce some measurement variability; three-dimensional or deep learning-assisted placental volumetry may provide more reproducible estimates [29]. Sixth, angiogenic biomarkers such as placental growth factor (PlGF) and soluble fms-like tyrosine kinase-1 (sFlt-1), which have demonstrated promising predictive performance in recent studies, were not available for analysis [30,31]. Finally, because the incidence of preeclampsia was relatively low, the confidence intervals of the corresponding estimates should be interpreted cautiously. Additionally, data on chronic hypertension, pregestational diabetes, prior preeclampsia, conception via assisted reproductive technology, maternal body mass index, gestational age at screening, and aspirin prophylaxis were not consistently available in this retrospective single-center dataset and could therefore not be incorporated as covariates in the multivariable models beyond maternal age, weight, parity, and smoking status; internal validation (leave-one-out cross-validation and bootstrap optimism correction) was applied to mitigate overfitting given the limited number of preeclampsia cases, but future prospective multicenter studies should incorporate these additional confounders and larger case numbers to enable more robust adjustment.

### 4.3. Clinical Implications

From a clinical perspective, the simplified two-dimensional estimation of placental volume is attractive because it is inexpensive, rapid, and can be integrated into the routine first-trimester scan without additional equipment or software. However, the present results indicate that, in a low-risk singleton population, neither placental volume nor the routinely available biochemical and Doppler markers—individually or combined—achieve the discriminatory accuracy required for stand-alone screening. These markers should therefore not be used in isolation to reassure or to triage low-risk women, and their apparent combined performance should not be reported without internal validation. The independent but weak association between low free β-hCG and fetal growth restriction may, at most, contribute modestly to future multimarker algorithms rather than serve as a stand-alone test. Early identification of these at-risk pregnancies remains clinically meaningful, given the well-established long-term cardiovascular consequences of fetal growth restriction for both mother and offspring [32].

### 4.4. Future Perspectives

Future research should focus on developing integrated prediction models that combine maternal characteristics, ultrasonographic parameters, established biochemical markers, and angiogenic factors such as PlGF and the sFlt-1/PlGF ratio [33]. Emerging approaches—including maternal serum metabolomic profiling, angiogenic factors, placental radiomics, and machine learning or artificial intelligence-based algorithms—may capture the biological complexity of placental dysfunction more effectively than any single marker [34,35]. Large prospective multicenter studies with external validation will be essential before such models can be reliably implemented in routine obstetric practice. Collectively, our findings suggest that future prediction strategies should move beyond isolated biochemical or ultrasonographic markers toward integrated multimodal models combining clinical variables, advanced imaging, angiogenic biomarkers, metabolomics, and artificial intelligence.

## 5. Conclusions

First-trimester placental volume, bilateral uterine artery Doppler pulsatility indices, maternal serum PAPP-A, and free β-hCG demonstrated limited predictive value for subsequent preeclampsia and fetal growth restriction in this retrospective cohort. Although lower maternal free β-hCG remained independently associated with fetal growth restriction after adjustment for maternal characteristics, the overall discriminatory performance of the individual biomarkers and their combined prediction model was insufficient for clinical application.

These findings indicate that currently available first-trimester biochemical and biophysical markers, when used individually, are unlikely to provide sufficient predictive performance for routine stand-alone screening of placental dysfunction. Future prediction strategies should incorporate multimodal approaches integrating clinical characteristics, advanced imaging, angiogenic biomarkers, metabolomics, and machine learning techniques to improve the early identification of pregnancies at increased risk for adverse placental outcomes. Future prospective multicenter studies incorporating angiogenic biomarkers, artificial intelligence-based image analysis, and external validation are warranted before these approaches can be recommended for routine clinical use.

## Figures and Tables

**Table 1:** Baseline maternal and pregnancy characteristics of the study population.

Characteristic	Value (*n* = 251)
Maternal age (years)	27.2 ± 5.3
Maternal weight (kg)	65.6 ± 12.5
Gravidity, median (range)	2 (0–6)
Parity, median (range)	0 (0–4)
Nulliparous, *n* (%)	127 (50.6)
Smoking, *n* (%)	23 (9.2)
Pre-existing maternal disease, *n* (%)	19 (7.6)
Cesarean delivery, *n* (%)	131 (52.2)
Preeclampsia, *n* (%)	8 (3.2)
Fetal growth restriction, *n* (%)	29 (11.6)

**Table 2:** Comparison of first-trimester biochemical and biophysical markers according to pregnancy outcome.

Marker	PE–No	PE–Yes	*p*	FGR–No	FGR–Yes	*p*
Placental volume (cm^3^)	80.1 [60.9–107.7]	68.0 [60.4–91.7]	0.478	80.2 [62.3–107.8]	74.3 [59.8–95.2]	0.342
Ut-A PI, right	1.59 [1.34–1.94]	1.78 [1.45–1.98]	0.499	1.59 [1.34–1.94]	1.67 [1.34–1.98]	0.628
Ut-A PI, left	1.63 [1.34–2.06]	1.71 [1.43–1.91]	0.757	1.64 [1.34–2.07]	1.60 [1.35–1.88]	0.620
Free β-hCG (MoM)	0.85 [0.60–1.38]	1.26 [0.64–2.17]	0.461	0.85 [0.62–1.42]	0.75 [0.48–1.07]	0.052
PAPP-A (MoM)	1.09 [0.77–1.56]	0.73 [0.47–1.41]	0.192	1.08 [0.76–1.55]	1.03 [0.69–1.73]	0.945

Values are median [interquartile range]. PE, preeclampsia; FGR, fetal growth restriction; PI, pulsatility index; MoM, multiple of median. *p*-values from the Mann–Whitney U test.

**Table 3:** Confounder-adjusted odds ratios (per 1-SD increment) of first-trimester biomarkers for preeclampsia (PE) and fetal growth restriction (FGR).

Marker	aOR (PE)	95% CI	*p*	aOR (FGR)	95% CI	*p*
Placental volume	0.71	0.30–1.71	0.449	0.81	0.53–1.26	0.350
Ut-A PI, right	1.26	0.62–2.54	0.522	1.04	0.71–1.53	0.838
Ut-A PI, left	1.02	0.49–2.14	0.950	0.88	0.53–1.45	0.610
Free β-hCG (MoM)	1.38	0.83–2.30	0.213	0.49	0.25–0.96	0.038
PAPP-A (MoM)	0.74	0.31–1.75	0.497	1.08	0.74–1.56	0.692

aOR, adjusted odds ratio (per 1-SD increment), adjusted for maternal age, weight, parity, and smoking; CI, confidence interval. Odds ratios are presented per one standard deviation increase in each biomarker. Corresponding areas under the ROC curve are shown in Figure 4.

## Data Availability

The raw data supporting the conclusions of this article will be made available by the authors upon request.

## Abbreviations

The following abbreviations are used in this manuscript:

FGR	Fetal growth restriction
UtA-PI	Uterine artery Doppler pulsatility index
PV	Placental volume
PAPP-A	Pregnancy-associated plasma protein-A
β-hCG	Beta-human chorionic gonadotropin
MoM	Multiple of median
ROC	Receiver operating characteristic
AUC	Area under the ROC curve
OR	Odds ratio
CI	Confidence interval
ACOG	American College of Obstetricians and Gynecologists
FMF	Fetal Medicine Foundation
PlGF	Placental growth factor
sFlt-1	Soluble fms-like tyrosine kinase-1
